# Chlorophyllin Inhibits Mammalian Thioredoxin Reductase 1 and Triggers Cancer Cell Death

**DOI:** 10.3390/antiox10111733

**Published:** 2021-10-29

**Authors:** Shibo Sun, Yici Zhang, Weiping Xu, Yue Zhang, Rui Yang, Jianli Guo, Shui Guan, Qiang Ma, Kun Ma, Jianqiang Xu

**Affiliations:** 1 School of Life and Pharmaceutical Sciences (LPS), Panjin Institute of Industrial Technology (PIIT), Liaoning Key Laboratory of Chemical Additive Synthesis and Separation (CASS), Dalian University of Technology, Panjin 124221, China; sunshibo@mail.dlut.edu.cn (S.S.); m15663671275_1@mail.dlut.edu.cn (Y.Z.); yangruirui@mail.dlut.edu.cn (R.Y.); jianli_guo@dlut.edu.cn (J.G.); makunonline@dlut.edu.cn (K.M.); 2 Interdisciplinary Research Center on Biology and Chemistry (IRCBC), Shanghai Institute of Organic Chemistry, Chinese Academy of Sciences, Shanghai 201210, China; zhangyici@sioc.ac.cn; 3 School of Ocean Science and Technology (OST), Dalian University of Technology, Panjin 124221, China; weiping.xu@dlut.edu.cn; 4 State Key Laboratory of Fine Chemicals, Dalian R & D Center for Stem Cell and Tissue Engineering, School of Chemical Engineering, Dalian University of Technology, Dalian 116023, China; guanshui@dlut.edu.cn; 5 Research & Educational Center for the Control Engineering of Translational Precision Medicine (R-ECCE-TPM), School of Biomedical Engineering, Faculty of Electronic Information and Electrical Engineering, Dalian University of Technology, Dalian 116024, China; 6 Chinese Academy of Inspection and Quarantine, Beijing 100176, China; maqiang@caiq.org.cn

**Keywords:** food colorants, thioredoxin reductase, chlorophyllin, cell death, selenocysteine, SecTRAPs (selenium compromised thioredoxin reductase-derived apoptotic proteins)

## Abstract

Food colorants are widely used by humans in food production and preparation; however, their potential toxicity requires an in-depth analysis. In this study, five out of 15 commercial food colorants, namely, lutein, betanin, caramel, crocin and chlorophyll, significantly inhibited wild type selenoprotein thioredoxin reductase 1 (TrxR1, TXNRD1) in vitro. The hyperactive Sec^498^ residue of TrxR1 was targeted by those five colorants, which was confirmed by the site-directed mutagenesis of TrxR1. Furthermore, two colorants, chlorophyll and betanin, triggered the oligomerization of TrxR1. A chlorophyll-derived compound, chlorophyllin, irreversibly inhibited the 5,5′-dithiobis-2-nitrobenzoic acid (DTNB) reducing activity of TrxR1 with *K*_inact_ = 6.96 × 10^−3^ ± 0.49 × 10^−3^ µM^−1^ min^−1^. Moreover, chlorophyllin reduced the cellular TrxR activity, leading to reactive oxygen species (ROS) accumulation and, subsequently, promoting cancer cell death. In conclusion, this study might contribute to understand the food safety of commercial colorants and provide chemotherapeutic compounds by targeting TrxR1.

## 1. Introduction

Food colorants are generally divided into synthesized and natural colorants, which are widely used in food processing, cosmetics production and chemicals synthesis [1]. Natural food colorants, such as anthocyanin, betaxanthin, betacyanin and β-carotene, and synthesized food colorants, including tartrazine, sunset yellow, and Ponceau 4R, are closely correlated to people’s life. Although the use of food colorant has strict regulations and standards, its potential toxicity is still a threat to the human body and health [2]. Recently, soda red was reported as being oversupplied to children, resulting in serious public food safety problems [3], and Azo compounds have been found to generate free radicals in cells [4]. Additionally, Red 40 and Yellow 6 facilitate the development of colitis in mice with increased IL-23 signalling [5]. Therefore, it is necessary to immediately evaluate the toxicity of food colorants and to search for their cell targets.

Selenoprotein thioredoxin reductase (TrxR, TXNRD) is essential for maintaining the redox states of thioredoxin (Trx, TXN) to exert its functions, such as regulating redox balance, supporting DNA synthesis, and resisting apoptosis signal [6,7]. Up-regulation of endogenous TrxR1 has been reported in several cancer cells, such as breast cancer and lung cancer, indicating an important role of TrxR1 in sustaining tumor phenotypes, and TrxR1 is regarded as a potential target for cancer chemotherapy [8]. It has been reported that chemotherapy drugs or natural plant-derived pigments blocked the highly reactive selenocysteine (Sec) residue of TrxR1, thereby triggering the selenium-compromised thioredoxin reductase-derived apoptotic proteins (SecTRAPs) mediated reactive oxygen species (ROS) accumulation and inducing cell death in several cancer cells [9,10]. Curcumin irreversibly modifies the Sec residue of TrxR1 and inhibits its reducing activity [11,12]. Juglone, a natural naphthoquinone secreted from the walnut tree (*Juglans regia* L.), is a Sec-independent substrate of TrxR1 and produces excessive superoxide radical in TrxR1-mediated redox recycling [13,14].

In this study, we assessed the effect of 15 widely used commercial food colorants on both isolated protein/enzyme and cultured cells. It was found that some colorants inhibited the TrxR1 activity and induced the oligomerization of TrxR1. Besides, a chlorophyll-derived compound, chlorophyllin, showed an anti-tumor capacity through inhibiting endogenous TrxR activity and triggering ROS production and accumulation in several cancer cell lines. This study is helpful for better understanding the application safety of food colorants and providing new insight into food colorants in cancer prevention and chemotherapy.

## 2. Materials and Methods

### 2.1. Materials

**Figure 1:**
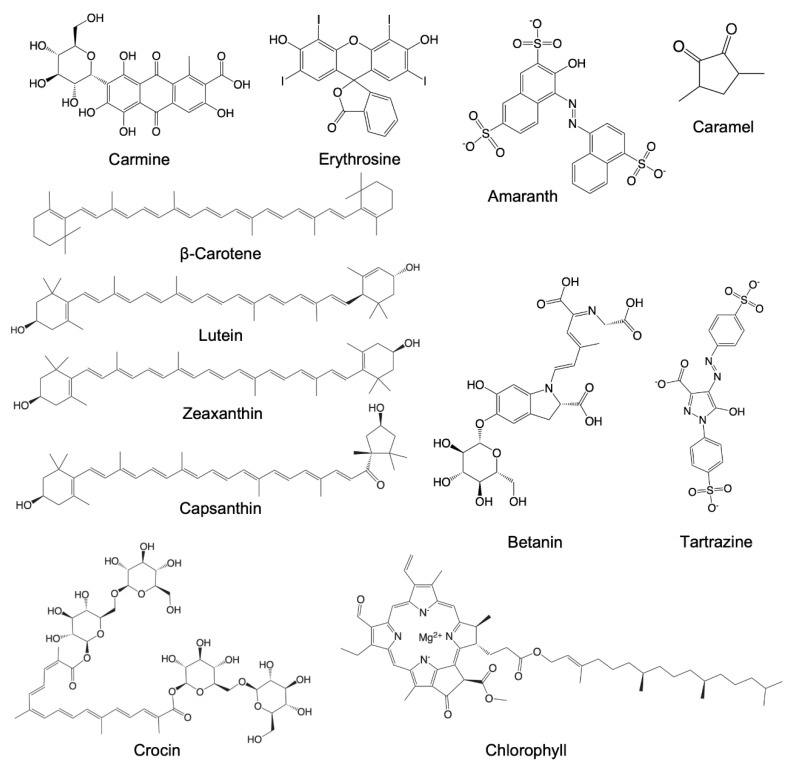
Chemical structures of food colorants. Some food colorants, including fruit-green, purple sweet potato color (PSPC) and sorghum red are commercially available, but are a mixture; therefore, the main structures of these food colorants were not shown.

The food colorants (as shown in Figure 1) were purchased from Bariren Biotechnology (Qingdao, China). Sodium copper chlorophyll (chlorophyllin) was bought from Aladdin (Shanghai, Chain). All food colorants were dissolved in DMSO to prepare a stock solution. NAP-5^TM^ desalting columns were purchased from Cytiva^TM^ (Uppsala, Sweden). RIPA buffer, including 50 mM Tris-HCl pH 7.5, 2 mM EDTA, 0.5% deoxycholate, 150 mM NaCl, 1% TritonX-100, 0.1% SDS, 1 mM Na_3_VO_4_ and 1 mM phenylmethanesulfonyl fluoride (PMSF), was purchased from Solarbio Science & Technology Co., Ltd. (Bejing, China). BCA protein assay kit was purchased from Sangon Biotech (Shanghai, China). Dulbecco’s modified eagle medium (DMEM) and heat-inactivated fetal bovine serum (FBS) were obtained from Gibco (Thermo Fisher Scientific, Waltham, MA, USA). 5,5′-dithiobis-2-nitrobenzoic acid (DTNB), 3-(4,5)-dimethylthiahiazo (-z-y1)-3,5-di-phenytetrazoliumromide (MTT) and ROS probe DCFH-DA were purchased from Sigma-Aldrich (St Louis, MI, USA). A 50 mM Tris-HCl containing 2 mM EDTA (pH 7.5) was used in all protein-based experiments, if not stated otherwise. Recombinant rat TrxR1 and its variants were column-purified using Äkta Start workstations (Cytiva^TM^, Uppsala, Sweden) as previously reported [15,16], and a good batch of wild type TrxR1 with specific activity of 22.5 U/mg in DTNB reduction was used for all enzyme activity assays unless otherwise stated.

### 2.2. TrxR1 Activity Assay

The enzymatic activities of TrxR1 and its various mutants were determined in parallel with DTNB and 9,10-panathrenequinone (9,10 PQ) as substrates [17]. In DTNB reduction assay, the working reaction mixtures contained 10 nM TrxR1, 2.5 mM DTNB and 300 µM NADPH in 50 mM Tris-HCl and 2 mM EDTA, pH 7.5. The formation of TNB^−^ anionic radicals was monitored at 412 nm for 10 min at room temperature, according to the linear region of the increase curves (assuming *Ɛ*_TNB_^−^, _412 nm_ = 13,600 M^−1^ cm^−1^). In the 9,10 PQ reduction assay, the working reaction mixtures contained 30 nM TrxR1, 30 µM 9,10 PQ and 200 µM NADPH, followed by NADPH oxidation at 340 nm for 20 min at room temperature, according to the linear region of the decrease curves (assuming *Ɛ*_NADPH_, _340 nm_ = 6200 M^−1^ cm^−1^).

### 2.3. NADPH Oxidase Activity Assay

NADPH oxidase activity of TrxR1 was measured by juglone reduction method [13,18]. The final reaction mixture consisted of 30 nM TrxR1, 30 µM juglone (in DMSO) and 200 µM NADPH. NADPH oxidation was measured in absorbance at 340 nm according to the linear decrease region of the decrease curves (assuming *Ɛ*_NADPH_, _340 nm_ = 6200 M^−1^ cm^−1^) for 30 min with 10 s interval at room temperature.

### 2.4. Cell Culture

Human HeLa, A549, HepG2, SH-SY5Y, MCF7 and AGS cells were cultured in DMEM containing 10% FBS, respectively. HCT116 cells were cultured in McCoy’s 5A medium (Procell, Wuhan, China) containing 10% FBS. The cells were cultured in medium containing 100 IU/mL penicillin, 100 µg/mL streptomycin (1% antibiotics) and 25 nM selenite at 37 °C in a humidified incubator with 5% CO_2_ (Thermo Fisher Scientific, Waltham, MA, USA).

### 2.5. Cell Viability Assay

MTT assay was used to detect cell viability. In brief, cells were seeded in 96-well plates with 5000 cells per well and allowed to stick overnight. The cells were then treated with chlorophyllin for 24 or 48 h. After treatment, the cell medium was carefully removed from the cultured cells and added with 100 µL fresh FBS-free medium containing 0.5 mg/mL MTT. The medium was incubated in an incubator for 4 h. Then, the medium was removed, and the formazan crystals formed were dissolved in 100 µL DMSO. The absorbance was measured at 570 nm, and 630 nm was set as the reference. The experiments were repeated four times. Regarding the auranofin treatment, cells were treated with auranofin (0 µM, 1 µM and 3 µM) for 2.5 h for inhibiting cellular TrxR1 activity; after incubation, auranofin was discarded and the fresh medium containing different concentrations of chlorophyllin were added. For N-acetyl-L-cysteine (NAC) treatment, cells were treated with different concentrations of chlorophyllin with or without NAC (0 µM, 100 µM and 500 µM) for 24 h; after incubation, the cell viability was determined.

### 2.6. Measurement of ROS Generation

Cells were placed at 40,000 cells per well into 6-well plates and treated with different concentrations of chlorophyllin for 12 h or for a specified time. After removing the medium, 10 µM DCFH-DA was added and incubated at 37 °C in darkness for 30 min. The cells were then observed and photographed using a Leica inverted fluorescence microscopy (Leica, Germany).

### 2.7. Cellular TrxR Activity Analysis with End-Point Insulin Assay

Cells were plated at 40,000 cells per well into 6-well plates and treated with different concentrations of chlorophyllin for 24 h or for a specified time. The cells were then collected and washed twice with ice-cold PBS. The total cell proteins were extracted with the RIPA buffer on ice for 30 min. The TrxR activities in the cell lysates were measured by the end-point Trx-coupled insulin assay [19] and the protein concentration was determined by BCA protein assay kit (Sangon, Shanghai, China), using bovine serum albumin (BSA) as protein standard.

### 2.8. Statistical Analysis

Data were represented as means ± S.E.M. Statistical significance was assessed by the Students’ *t*-test. Compare means between control and treatment groups were calculated using one-way ANOVA analysis by GraphPad Prism 8.0 (San Diego, CA, USA). * *p* < 0.05, ** *p* < 0.01, *** *p* < 0.001, and n.s. means not significant.

## 3. Results

### 3.1. None of 15 Food Colorants Are Substrates of the Selenoprotein TrxR1

To analyze the potential effects of common food colorants on TrxR1, we selected 15 commercial food colorants, including crocin, caramel, betanin, chlorophyll, lutein, amaranth, β-carotene, capsanthin, carmine, erythrosine, fruit-green, purple sweet potato color (PSPC), sorghum red pigment (SRP), tartrazine and zeaxanthin (food-grade, Figure 1). We first wanted to know whether these food colorants are good substrates of TrxR1. The results showed that these food colorants did not react with NADPH, and none of them exhibited as a proper substrate to be reduced by TrxR1 (data not shown).

### 3.2. Caramel, Crocin, Betanin, Chlorophyll and Lutein Inhibit the TrxR1 Activities

**Figure 2:**
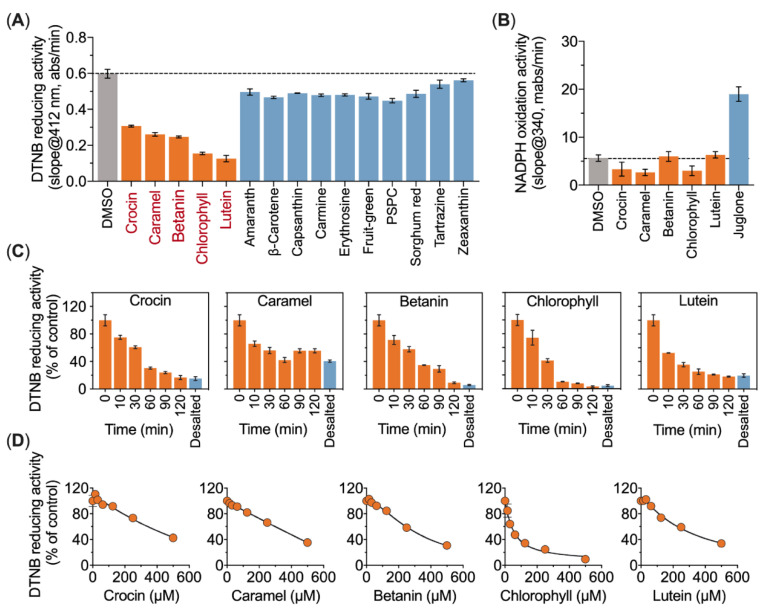
Food colorants inhibit TrxR1 activity. (**A**) Food colorants inhibit the DTNB reducing activity of TrxR1. TrxR1 was pre-reduced by 100 µM NADPH for 10 min and incubated with various food colorants (500 µM) for 30 min in dark. Subsequently, the enzyme activity was determined by DTNB reducing assay. (**B**) NADPH consumption of TrxR1 with 30 µM selected food colorants. Juglone was used as the positive control. (**C**) Time-dependent inhibition of TrxR1 by food colorants. NADPH pre-reduced TrxR1 was incubated with 500 µM food colorants, and the enzyme activity was determined at indicated times. After incubation for 2 h, the enzyme was desalted using a NAP-5^TM^ column. (**D**) Dose-dependent inhibition of selected food colorants on TrxR1 mediated DTNB reduction.

Next, we found that five of the 15 food colorants, including crocin, lutein, betanin, caramel and chlorophyll, significantly inhibited the TrxR1 activity (Figure 2A). Meanwhile, compared with juglone, none of these five food colorants were good substrates of TrxR1 (Figure 2B). To reveal the inhibition mechanism, we incubated the enzyme with the five food colorants separately for predefined period of time. The results showed that crocin, betanin, lutein and chlorophyll inhibited TrxR1 in a time-dependent manner. Notably, the inhibtion of TrxR1 by the five food colorants could not be rescued after desalting, indicating an irreversible inhibition occurred (Figure 2C). Finally, the IC_50_ values of the selected food colorants on TrxR1’s DTNB reducing activity were determined, and chlorophyll showed a strong inhibition with IC_50_ = 48.55 µM, lutein with IC_50_ = 306.10 µM, crocin with IC_50_ = 776.60 µM and betanin with IC_50_ = 323.10 µM. In addition, it is not applicable for determining the IC_50_ values of caramel due to it ambiguous fitted curve (Figure 2D).

### 3.3. Food Colorants Selectively Target TrxR1’s Highly Reactive Sec^498^ Residue Rather Than Catalytic Cys Residues

**Figure 3:**
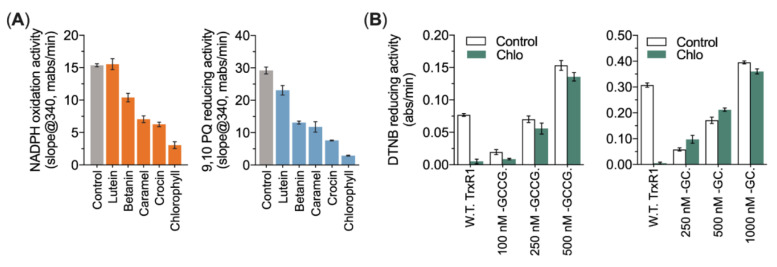
Identification of the Sec^498^ residue of TrxR1 targeted by chlorophyll. (**A**) Food colorants inhibited the juglone reducing activity and the 9,10 PQ reducing activity of TrxR1. TrxR1 was pre-reduced with 100 µM NADPH for 10 min, and then, 500 µM different food colorant were incubated with TrxR1 in dark for 30 min, respectively. Subsequently, the TrxR1 activity of juglone and 9,10 PQ as substrates was determined. (**B**) Chlorophyll has no inhibition on either TrxR1 Sec-to-Cys mutant or the UGA-truncated mutant. Wild type TrxR1, GCCG variants (1 µM, 2.5 µM, 5 µM) and truncated TrxR1 (2 µM, 5 µM, 10 µM) were incubated with 500 µM Chlorophyll for 60 min. After incubation, the enzyme activity was determined by using DTNB as substrate.

The inhibitory effects of various food colorants on NADPH oxidation activity and 9,10 PQ reducing activity of TrxR1 were tested next. It was found that lutein had no inhibition on the NADPH oxidation activity of TrxR1, and the inhibitory effect on the 9,10 PQ reduction was not as good as other food colorants. Surprisingly, chlorophyll strongly inhibited the 9,10 PQ reducing activity of TrxR1, remaining only <5% activity (Figure 3A). Therefore, the possible amino acid residues of TrxR1 targeted by chlorophyll were further detected. The results demonstrated that either the U498C mutant (-GCCG) or the UGA-truncated TrxR1 mutant (-GC), were insensitive to chlorophyll (Figure 3B), indicating that the reactive C-terminal Sec residue of TrxR1 is the primary target of chlorophyll.

### 3.4. Chlorophyll and Betanin Induce Covalent Crosslinks between TrxR1 Subunits

**Figure 4:**
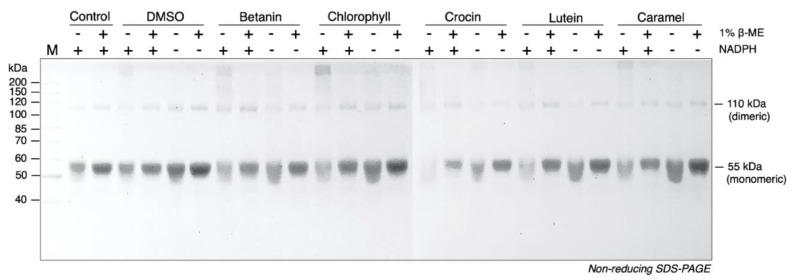
Chlorophyll and betanin trigger the oligomerization on wild type TrxR1 in vitro. Wild type TrxR1 (0.8 µM) was incubated with 500 µM food colorants for 24 h at room temperature in dark with or without 200 µM NADPH. DMSO was used as the control. After incubation, samples were subjected to 10% non-reducing SDS-PAGE, followed by Coomassie Blue staining, and documented using ChampGel^TM^ 5000 Plus gel imaging system (Sage Creation Science Corp., Beijing, China).

We found that the NADPH-reduced TrxR1 formed multimers after being treated with 500 µM chlorophyll or betanin for 24 h at room temperature, which showed about 220 kDa on a non-reducing SDS-PAGE (Figure 4). The TrxR1 multimers could be converted into monomer in the presence of 1% β-mercaptoethanol (β-ME), suggesting a crosslinker formed between TrxR1 subunits upon colorant treatment.

### 3.5. Chlorophyllin Inhibits TrxR1 in a Time- and Dose-Dependent Manner

**Figure 5:**
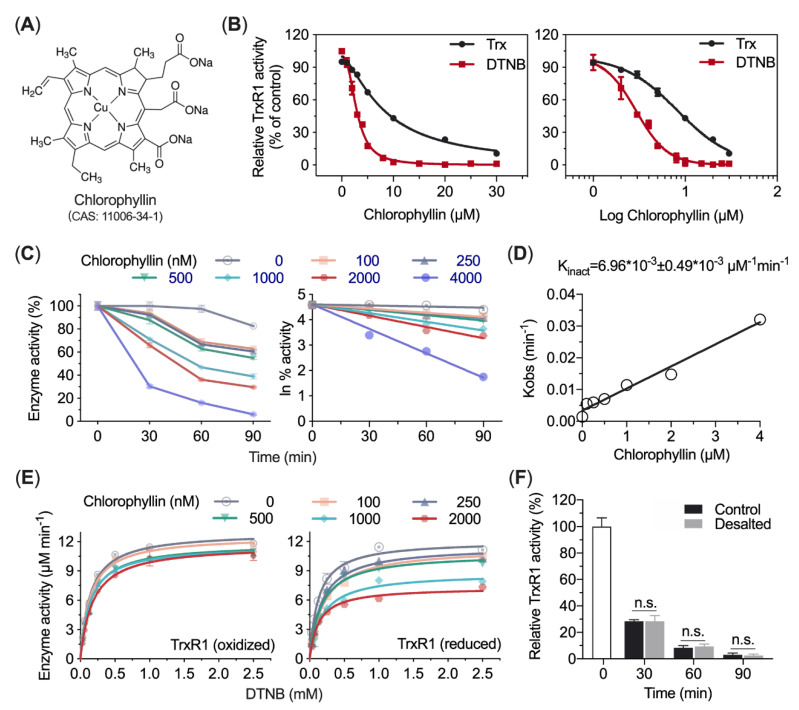
Inhibitory effect of chlorophyllin on TrxR1 enzymatic activity and kinetic behavior. (**A**) Chemical structure of chlorophyllin. (**B**) Chlorophyllin inhibits both DTNB reduction activity and Trx-coupled insulin reduction activity of TrxR1. (**C**) Time-dependent inhibition of TrxR by chlorophyllin. TrxR1 was incubated with different concentrations of chlorophyllin for different times, and the enzyme activity was determined with DTNB as substrate, which was normalized with the untreated enzyme. The remaining activity of TrxR1 was converted to Napierian logarithm, and the *K*_obs_ and *K*_inact_ were shown in (**D**) with chlorophyllin ranging from 0 to 4 µM. (**E**) Chlorophyllin inhibits reduced TrxR1 rather than oxidized TrxR1. TrxR1 was pre-reduced by NADPH or not and incubated with different concentrations of chlorophyllin for 1 h. Subsequently, the enzyme activity was tested by DTNB reducing assay. (**F**) The inhibition of chlorophyllin on TrxR1 is irreversible. 0.5 µM TrxR1 was incubated with 3 µM chlorophyll, and the free chlorophyll was removed by NAP-5^TM^ column (Cytiva^TM^, Uppsala, Sweden) at the indicated time. The enzyme activity was determined by using DTNB as a substrate. n.s. means not significant.

To further determine the interaction between chlorophyll and TrxR1, an analytically pure sodium copper chlorophyll also called chlorophyllin, was used in this experiment (Figure 5A). When NADPH-reduced TrxR was incubated with chlorophyllin for 30 min, its DTNB reduction activity and Trx reduction activity were lost (Figure 5B). The IC_50_ values of chlorophyllin were 2.65 µM in DTNB reduction and 9.17 µM in Trx-coupled insulin reduction, respectively. Chlorophyllin showed a time-dependent inhibition on TrxR1, and the DTNB reducing activity of TrxR1 was inhibited at a second-order rate of 6.96 × 10^−3^ µM^−1^ min^−1^ (Figure 5C,D). It was also found that oxidized TrxR1 was insensitive to chlorophyllin compared with reduced TrxR1 (Figure 5E). This is consistent with the fact that only reduced TrxR1 containing the free selenocysteines, which is more susceptible to chlorophyll attack at micromolar concentration. Clearly, this experiment showed that chlorophyllin inhibited TrxR1, which was enhanced along with the time. After desalting the samples at indicated incubation times, the enzyme activity was not recovered but remained pretty the same level stably, suggesting the inhibition of chlorophyllin to TrxR1 is irreversible (Figure 5F).

### 3.6. Chlorophyllin Causes Cellular ROS Accumulation

**Figure 6:**
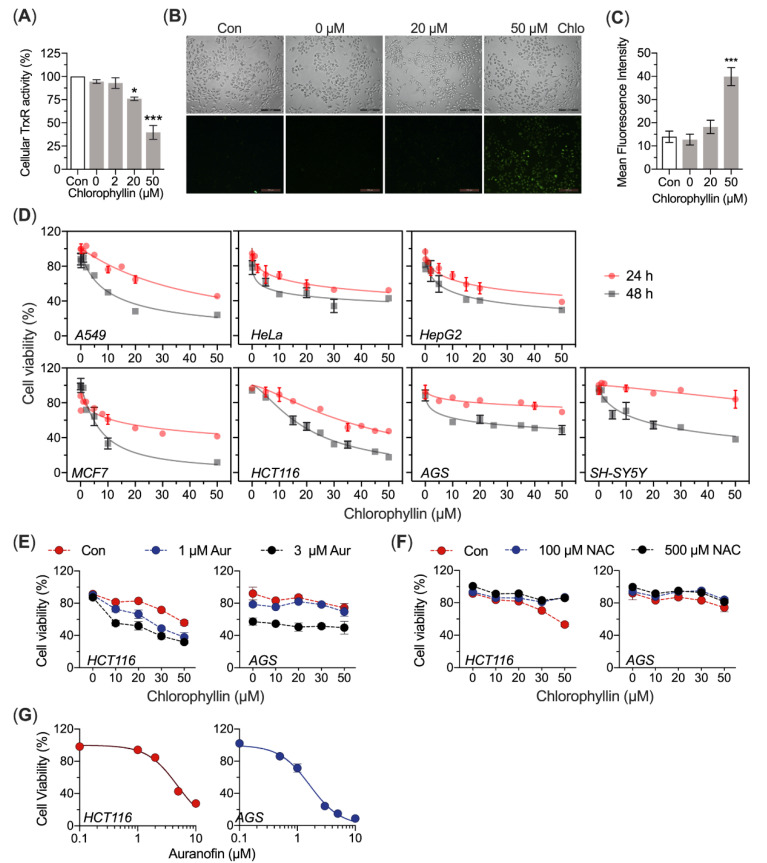
Chlorophyllin inhibits the cellular TrxR activity by using cancer cell lines. (**A**) Chlorophyllin inhibits the cellular TrxR activity. HeLa cells were harvested by using RIPA buffer on ice, then the cellular TrxR activity was determined by end-point Trx-coupled insulin assay and the protein concentration was determined using a BCA kit. (**B**) Chlorophyllin induces ROS production in HeLa cells. HeLa cells were treated with chlorophyllin for 12 h. After incubation, the cellular ROS were detected by using DFCH-DA. Phase-contrast (top) and fluorescence (bottom) images were acquired by fluorescence microscopy. The mean fluorescence intensities from (**B**) were quantified by ImageJ^TM^ (NIH, USA) and shown in (**C**) with the treatment of chlorophyllin up to 50 µM. (**D**) The cell viability was determined by MTT. Chlorophyllin induces cancer cell death by inhibiting TrxR1. Human A549 cells, HeLa cells, HepG2 cells, MCF7 cells, HCT116 cells, AGS cells and SH-SY5Y cells were incubated with chlorophyllin at the indicated concentrations for 24 h and 48 h, respectively. (**E**,**F**) Cell viability of HCT116 cells and AGS cells exposed to chlorophyllin alone or in combination with auranofin (2.5 h pre-treatment) or NAC (24 h co-treatment). (**G**) Cytotoxicity of auranofin on HCT116 cells and AGS cells. Cells were treated with auranofin for 2.5 h, and the cell viability was determined using MTT assay. (* *p* < 0.05 and *** *p* < 0.001).

To investigate the effects of chlorophyllin on cellular TrxR, the cellular TrxR activity in chlorophyllin-treated HeLa cells was determined. The results showed that chlorophyllin treatment caused remarkable inhibition of cellular TrxR1 activity in HeLa cells compared with untreated controls (Figure 6A). Since TrxR1 acts as an antioxidant enzyme, ROS levels in HeLa cells treated with chlorophyllin were then measured. Fluorescent signals of ROS probe DCFH-DA were much higher after chlorophyllin treatment, suggesting that chlorophyllin can cause oxidative stress via inhibiting endogenous TrxR activity (Figure 6B,C).

### 3.7. Chlorophyllin Leads to Cell Death

Since food colorants are consumed orally, we investigated the cytotoxicity of chlorophyllin on colorectal cancer cell line HCT116 and gastric carcinoma cancer cell line AGS, Compared with AGS cells, HCT116 cells were more sensitive to chlorophyllin (Figure 6D). We also determined the cytotoxicity of chlorophyllin towards a various cancer cell line, including human HeLa cells, A549 cells, HepG2 cells, MCF7 cells and SH-SY5Y cells, results showed that chlorophyllin possessed potent cytotoxicity towards cancer cells in dose- and time-dependent manners (Figure 6D). The IC_50_ values of chlorophyllin on cancer cell lines are shown in Table 1. We found that pre-treated with auranofin (TrxR1 inhibitor) to inhibit TrxR activity, increasing the sensitivity of HCT116 cells to chlorophyllin, suggesting that the cytotoxicity of chlorophyllin was related to TrxR1. In contrast, 100 µM or 500 µM NAC protect cell from chlorophyllin in both HCT116 cells and AGS cells (Figure 6E,F). The cytotoxicity of auranofin on HCT116 cells and AGS cells is shown in Figure 6G.

## 4. Discussion

Food colorants are widely used in the human diet, but some are potentially toxic and having side effects on humans; therefore, their toxicological and pharmacological properties should be carefully addressed. Mammalian selenoprotein TrxR1 is a well-known antioxidant enzyme whose antioxidant activity is completely dependent on a highly active C-terminal Sec residue, which is easily destroyed by antitumor drugs or environmentally contaminated electrophiles. Thus, two urgent questions need to be addressed: (1) Can food colorants induce oxidative stress through the interaction with the Trx system in cell? And (2) How do food colorants display toxicity or side effects by targeting the Trx system? To answer these questions, a condensed study was conducted; surprisingly, it found that some colorants are good TrxR1 inhibitors, which limited the safety applications of food colorants, but provided new strategies that can contribute to targeting Trx systems for clinical chemotherapy.

To determine the inhibition effects of food colorants on TrxR1 in vitro, 15 food colorants with different structures were selected, including carotenoids (lutein, zeaxanthin, capsanthin, β-carotene, crocin), azo pigment (tartrazine, amaranth), anthocyanin (purple sweet potato), betalains (betanin), porphyrin (chlorophyll) and fruit green, caramel, sorghum red, capsanthin and erythrosine (Figure 1). Surprisingly, several food-grade pigments were found to irreversibly inhibit TrxR1 reducing activity, including betanin, chlorophyll, caramel, lutein and crocin (Figure 2). The previous study has shown that artificial food coloring is related to child hyperactivity and promotes colitis in mice [2]. However, in this study, these food colorants did not inhibit the TrxR1 activity (Figure 1).

Chlorophyll, as one of the most abundant biomolecules on earth, has been found to be associated with human health [20]. For instance, chlorophyll reduces aflatoxin DNA adducts and decreases the risk of colon cancer in males [21,22]. In this study, chlorophyllin inhibited TrxR1 activity, with the *K*_inact_ of 6.96 × 10^−3^ ± 0.49 × 10^−3^ µM^−1^ min^−1^ in DTNB reduction (Figure 5). In the previous study, Nielsen et al. found that PpIX and its derivatives, NMPP and hemin, are TrxR1 inhibitors using high throughput screening (HTS). The *K*_inact_ values of PpIX, NMPP and hemin in TrxR1 mediated Trx-coupled insulin reduction is 0.30 × 10^−3^ µM^−1^ min^−1^, 0.73 × 10^−3^ µM^−1^ min^−1^ and 0.26 × 10^−3^ µM^−1^ min^−1^, respectively, indicating that some porphyrin compounds inhibit TrxR1 in a time-dependent manner [23]. In addition, IC_50_ values of chlorophyllin inhibiting TrxR1′s DTNB reducing and Trx reducing activity were also determined, with 2.65 µM and 9.17 µM, respectively, indicating that chlorophyll inhibited TrxR1 in a dose-dependent manner. The apparent *K*i value determined by Nielsen et al. in the inhibition of PpIX on TrxR1′s Trx reduction is 2.7 µM. However, in this study, the *K*i value of chlorophyllin on TrxR1 was not applicable because its apparent *K*m values did not change versus different concentrations of chlorophyllin (Data not shown). 

The inhibition of TrxR1 by chlorophyll (or chlorophyllin) may be related to its biological function [23]. Chlorophyllin has also been reported to decrease the expression and activity of Nrf2-mediated heme oxygenase-1 [24]. Meanwhile, the loss of TrxR1 activity causes induction of Nrf2 and hepatic hemeoxygenase-1 [25]. These findings indicate that TrxR1 may act as a molecular target for chlorophyllin or porphyrin compound. Significantly, pre-inhibiting TrxR1 activity by auranofin makes cancer cells sensitive to chlorophyllin (Figure 6E,F). Previous studies showed that knockdown of TrxR1 or pharmacological inhibition of TrxR1 could yield drug-specific alteration of cytotoxicity of therapeutic small molecules [26], which also suggested that the TrxR1 activity was related to the biological function of chlorophyll (or chlorophyllin).

Recently, Che et al. found that gold(III) mesoporphyrin IX form thiol adducts with cysteines of Trx and peroxiredoxin (Prx) via the meso-carbon atom of the porphyrin ligand and displays effective in vitro and in vivo anticancer activity [27]. Our findings in this research were consistent with the previous study, demonstrating that porphyrin compounds modified the cysteine or selenocysteine of TrxR1 and inhibited its activity.

The Trx system is considered to be the central antioxidant system of the cells and up-regulated Trx system in cancer cells-facilitated tumor’s phenotype and metastasis maintenance [28,29,30,31,32]. Targeting Trx system may lead to the production and accumulation of excessive ROS, which is an attractive strategy in cancer treatment [33,34,35,36]. TrxR contains a highly active and surface-exposed selenocysteine-containing motif, which is the key redox-active motif that reduces Trx or thioredoxin-related protein of 14 kDa (TRP14) and is considered to be a drug target [36,37,38]. Over the past few decades, many drugs have been reported as inhibitors of TrxR1 [39,40,41,42,43]. Some of them modify the C-terminal Sec residues of TrxR, and convert the enzymes from antioxidant enzymes to NADPH oxidases by SecTRAPs, including pigments such as juglone and curcumin [18].

Additionally, reduced levels of Trx1 can inhibit apoptosis signal-regulated kinase (ASK-1). Activity loss of TrxR1 causes Trx1 oxidation, leading to AKS-1 release and apoptosis [44]. Besides, Trx1 is essential for ribonucleotide reductase (RNR) activity, and pharmacological inhibit TrxR1 activity may cause replication stress [45]. In this study, it was found that chlorophyll inhibited TrxR1 activity in a dose- and time-dependent manner, so we investigated the potential anti-tumor properties of chlorophyllin in several cancer cells (Figure 6). Additionally, we found that chlorophyllin inhibited glioma cell line SH-SY5Y; however, some in vivo studies should be investigated in the future to discuss the capacity of chlorophyllin delivered to brain tumors.

The major form of active TrxR1 is a non-covalently linked homodimer, and each subunit of TrxR1 dimer is 55 kDa. Oligomerization of TrxR1 was first found in human T cells [46] and described in kinetic [47] and crystal [48]. The formation of TrxR1 oligomers is caused by the oxidation of the surface-exposed Trp^114^ residues of TrxR1 in response to oxidative stress. Interestingly, some low-weight compounds can also make cross-links between two TrxR1 subunits, such as cisplatin [49] and *p*-benzoquinone [50]. In this study, chlorophyll induced the oligomerization of NADPH pre-reduced TrxR1, and the oligomerization bands disappeared after treating with 1% β-ME. It is speculated that the TrxR1′s oligomer is caused by the superoxide anion radicals induced the covalent link between two subunits (Figure 4).

## 5. Conclusions

In summary, we found that food colorants including lutein, betanin, caramel, crocin and chlorophyll irreversibly inhibited TrxR1 activity by targeting the selenocysteine residues. Chlorophyll and betanin induced TrxR1’s oligomerization. We also demonstrated that chlorophyllin is a new inhibitor of TrxR1 that induces ROS accumulation and cancer cell death. The obtained data are helpful in revealing the interaction between food colorants and selenoprotein TrxR1, and assist in better understanding the toxicity and safe application of food colorants.

## Figures and Tables

**Table 1:** IC_50_ values (µM) of chlorophyllin on cancer cell lines.

Time (h)	A549	HeLa	HepG2	MCF7	HCT116	AGS	SH-SY5Y
24 h	40.7 ± 3.9	47.9 ± 13.5	36.8 ± 11.2	30.1 ± 7.1	43.5 ± 2.6	n.a.	n.a.
48 h	10.3 ± 1.4	10.4 ± 4.3	10.2 ± 3.0	6.9 ± 1.1	20.2 ± 0.8	49.6 ± 24.7	31.7 ± 6.7

Note: n.a., not applicable.

## Data Availability

The data presented in this study are available in article.
